# HedgeRank: Heterogeneity-Aware, Energy-Efficient Partitioning of Personalized PageRank at the Edge

**DOI:** 10.3390/mi14091714

**Published:** 2023-08-31

**Authors:** Young-Ho Gong

**Affiliations:** School of Software, Soongsil University, Seoul 06978, Republic of Korea; yhgong@ssu.ac.kr

**Keywords:** personalized PageRank, graph processing, energy efficiency, edge devices, heterogeneous processing units

## Abstract

Personalized PageRank (PPR) is a widely used graph processing algorithm used to calculate the importance of source nodes in a graph. Generally, PPR is executed by using a high-performance microprocessor of a server, but it needs to be executed on edge devices to guarantee data privacy and network latency. However, since PPR has a variety of computation/memory characteristics that vary depending on the graph datasets, it causes performance/energy inefficiency when it is executed on edge devices with limited hardware resources. In this paper, we propose *HedgeRank*, a heterogeneity-aware, energy-efficient, partitioning technique of personalized PageRank at the edge. *HedgeRank* partitions the PPR subprocesses and allocates them to appropriate edge devices by considering their computation capability and energy efficiency. When combining low-power and high-performance edge devices, *HedgeRank* improves the execution time and energy consumption of PPR execution by up to 26.7% and 15.2% compared to the state-of-the-art PPR technique.

## 1. Introduction

Personalized PageRank (PPR) is a variant of PageRank that is widely used in graph-based neural networks, recommendation systems, social network analysis, image processing, bioinformatics, etc. [1,2,3,4,5,6]. PageRank calculates the importance of each node for all nodes in a graph, whereas PPR calculates the importance and relevance of each node for the source node. In general, PPR is used to calculate the rank of some nodes (e.g., top-*k* nodes) whose score is higher than that of all the other nodes combined. Previously, though PPR has been executed on server systems due to its large memory requirement, it has recently moved to edge devices to secure data privacy with a lower network cost [7].

However, as the graph size has exploded significantly, edge devices suffer from a lack of memory to execute graph processing such as PPR; for example, a graph dataset from Facebook consists of 1.39B nodes and 400B edges [8]. In addition to the memory size problem, PPR causes complex memory access patterns due to the sparsity of the graph dataset, which degrades performance as well as the effective memory bandwidth [9,10].

Many researchers have made efforts to solve the overhead of graph processing based on various software approaches [11,12,13,14,15], but they still use a large memory size. Furthermore, though researchers have also proposed graph-processing accelerators [9,16,17,18,19], such a fixed hardware accelerator, they cannot provide flexibility in graph processing. For instance, when the graph size is larger than the size of the on-chip memory of an accelerator, the accelerator may suffer from serious performance degradation due to excessive off-chip memory accesses.

In this paper, we propose *HedgeRank*, an energy-efficient PPR partitioning technique for edge devices with heterogeneous processing units. Unlike the previous studies mentioned above (e.g., software modifications [14,15] or hardware accelerators [9,19]), our *HedgeRank* exploits existing processing units (i.e., CPUs and GPUs) in real-world edge devices to enhance the energy-efficiency of PPR execution. Since the state-of-the-art edge devices have heterogeneous processing units with different computing powers and memory specifications, we need to partition the conventional PPR into several subprocesses to be executed on an appropriate processing unit among edge devices, depending on the size of the input graph and computation/memory usage characteristics. *HedgeRank* consists of three subprocesses as follows: (1) the breadth-first-search-based load balancing, (2) the subgraph partitioning, and (3) the calculation of the importance and relevance. Note that, though Li et al. [16] also proposed a similar PPR partitioning technique with FPGA acceleration called MeLoPPR (Memory-Efficient, Low-Latency PPR), MeLoPPR still causes significant performance overhead in general processing units (e.g., CPUs) as the number of next-stage nodes increases. Moreover, since MeLoPPR exploits FPGA hardware acceleration, it is not flexible/adaptive to the size of graph and also causes significant costs when the size of graph increases significantly. Contrary to the MeLoPPR, our *HedgeRank* is much more flexible with the size of the graph, while providing better energy efficiency for edge systems. Our experiment on off-the-shelf edge devices (combining low-power and high-performance edge devices) shows that *HedgeRank* improves the performance of PPR execution by up to 26.7%, even with a lower energy consumption (up to 15.2%), compared to MeLoPPR.

## 2. Background and Related Work

### 2.1. Personalized PageRank (PPR)

PageRank computes the relevance or importance of each node in a graph under a random surfer model governed by a teleportation parameter. PageRank can be interpreted as the stationary distribution of a random walk on a graph that restarts from a uniform location at each time with probability α ∈ (0, 1). Meanwhile, personalized PageRank (PPR) is a personalized version of the PageRank algorithm for scoring the relevance of graph nodes to a set of source nodes based on the random walk with restart. Contrary to the random walk used in the conventional PageRank algorithm, which can go to any of the adjacent nodes, the random walk with restart used in PPR restarts on a specific restart node.

Though PPR is used in a wide range of fields, the graph dataset has become massive, meaning that PPR suffers from large memory requirements. Furthermore, the poor locality of graph datasets causes irregular memory access patterns in PPR, which makes PPR worse in terms of energy efficiency as well as performance.

### 2.2. Software-Based Performance Improvement Techniques for PPR

To solve the performance overhead of PPR, researchers have proposed advanced PPR algorithms to optimize memory access patterns and reduce the execution time. Bahmani et al. proposed Monte Carlo algorithms to reduce the PPR execution time [15]. The Monte Carlo algorithm exploits short random walk segments starting at each node in the graph. Wei et al. proposed TopPPR to compute the top-*k* entries up to a user specified precision [12]. TopPPR minimizes the accuracy loss through random walk sampling, forward search, and backward search in large graphs and also ensures accuracy. Lofgren et al. proposed FAST-PPR using the basic Monte Carlo method [13]. FAST-PPR reduces the computation complexity from O(δ) to Ω(1/δ). Shi et al. proposed kPAR to perform the PPR queries in real time based on parallelization and acceleration with the GPU [14]. In addition to software PPR acceleration techniques, Li et al. recently proposed EvePPR to solve the problems of dynamic and evolving graph characteristics in PPR applications [20]. Although the above studies have improved the conventional PPR while maintaining a certain level of accuracy (i.e., the precision of top-*k* entries), they did not consider the memory overhead and thus still suffer from a lot of memory usage. Since memory consumes a large portion of energy, such software techniques are not enough to improve energy efficiency.

### 2.3. Hardware-Based, Energy-Efficient PPR

In addition to the software modification to improve performance, researchers have proposed graph processing accelerators based on processing-in-memory (PIM) architectures, which provide much higher performance with lower memory overhead compared to conventional processing units. Ham et al. proposed Graphicionado, a graph processing accelerator design that consists of on-chip memory for storing vertices of graph datasets and a specialized data path to achieve better performance and lower energy consumption than the conventional microprocessors [18]. Dai et al. proposed another PIM architecture, called Graphh based on a hybrid memory cube (HMC) [9]. Graphh improves performance with load balancing on the HMC. Song et al. proposed GraphR based on ReRAM-based analog computing, which reduces memory access overhead by optimizing memory access and configuring a computing logic circuit around memory, resulting in a shorter execution time and lower energy consumption compared to other PIM-based architectures [19].

Though such hardware accelerators could be exploited to reduce PPR execution time by mitigating memory overhead or exploiting specialized processing units, they may not be flexible to various graph datasets. For example, an accelerator may suffer from excessive off-chip memory accesses when a graph’s data does not fit into its on-chip memory. In the case of graph processing techniques such as PPR, the graph datasets have various characteristics (e.g., number of nodes, node connectivity, etc.). Furthermore, the graph data could morph at runtime (e.g., node addition/deletion). Thus, flexibility is quite important for graph processing. A lack of flexibility causes energy inefficiency since it causes the underutilization of each hardware unit; thus, such techniques inevitably require more hardware units (e.g., PIM arrays, processing units, or more LUTs) as well as a larger memory capacity to cover various data characteristics. Due to the reason, even though many advanced techniques have been proposed, PPR is still widely used with general processing units (e.g., CPUs or GPUs) in real-world applications [21,22,23,24].

### 2.4. Memory Optimization Techniques for PPR

**Figure 1:**
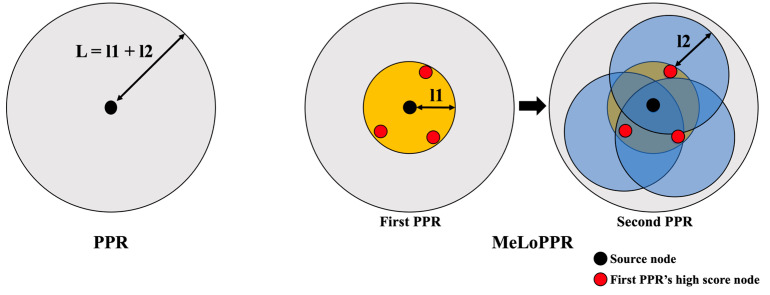
An illustration of PPR and MeLoPPR (reproduced from [16]).

Li et al. proposed MeLoPPR (Memory-efficient, Low-latency PPR), which divides the PPR operations into two stages to compute the PPR with a lower memory overhead [16]. Figure 1 shows a diagram of the conventional PPR and MeLoPPR.

As shown in the Figure 1, when the length of a graph is *L*, and the conventional PPR calculates scores for all the nodes within *L* (i.e., all the nodes in the graph) in a single stage. On the other hand, as shown in the Figure 1, MeLoPPR calculates not the entire graph in a single stage but a subset of the graph by dividing the length *L* into *l*1 and *l*2 (*L* = *l*1 + *l*2). In emerging applications, such as recommendation systems, the highest top-*k* nodes are more important than the other nodes. Thus, MeLoPPR only exploits a subset of the graph by dividing the conventional PPR into two stages. MeLoPPR first performs PPR computation on the subgraph from the source node to the nodes within the length *l*1 (i.e., the first PPR). After the first PPR, several high-score nodes are selected as source nodes of the second PPR. In the second PPR, MeLoPPR computes the scores of the nodes within the length *l*2 (from each selected node). Though MeLoPPR provides slightly better flexibility than ASIC accelerators such as Graphicionado, it causes much higher cost (i.e., more FPGA LUTs or BRAMs) for supporting a larger graph size or higher parallelism.

## 3. Energy-Efficient Partitioning of Personalized PageRank for Heterogeneous Edge Devices 

### 3.1. Motivation

**Figure 2:**
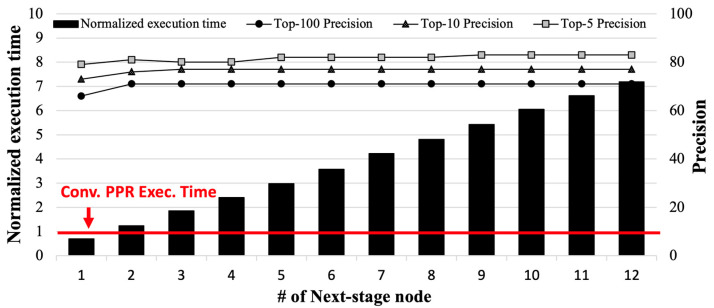
Execution time and top-*k* precision of MeLoPPR compared to the conventional PPR, using com-youtube graph. The execution time is normalized to the conventional PPR.

As a motivational experiment, we evaluated the performance and energy efficiency of the conventional PPR and the state-of-the-art PPR technique (i.e., MeLoPPR by Li et al.) [16] on real-world edge devices (i.e., Nvidia Jetson AGX Xavier, Santa Clara, CA, USA) [25]. Figure 2 shows its execution time and top-*k* accuracy (i.e., *k* = 5, 10, and 100) compared to the conventional PPR, depending on the number of second-stage nodes of MeLoPPR; the details on our experimental environment will be described in Section 4.1.

As shown in Figure 2, MeLoPPR causes performance overhead as the number of second-stage nodes increases, compared to the conventional PPR. According to our analysis, the top-*k* accuracy is saturated when the number of second-stage nodes is larger than five, while the execution time increases linearly. Since MeLoPPR only exploits CPU cores on the edge device, it shows inefficiency in the tradeoff between performance and top-*k* accuracy. Since recent edge devices have heterogeneous processing units with different memory specifications, PPR computation needs to be carefully partitioned into appropriate processing units in edge devices for better performance and energy efficiency.

**Figure 3:**
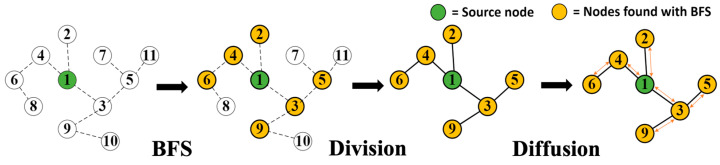
Subprocesses of the conventional PPR.

Figure 3 shows the subprocesses of the PPR. Note the initial numbers of each node in Figure 3 are assigned randomly. 

We describe each subprocess of the PPR shown in Figure 3 as follows. First, the BFS (Breadth-First-Search) traverses the graph from a specific source node to identify its adjacent nodes. Second, the Division process analyzes the connection between the nodes to form a subgraph consisting of the source node and the nodes within a certain distance. Lastly, the Diffusion process calculates the PageRank scores of the nodes by performing a random walk with restart on a subgraph created by the Division process and adds the calculated PageRank scores to the entire score only when performing a second PPR. As the BFS and Division process search and connect the nodes, respectively, the execution time of the processes are proportional to the number of nodes. On the other hand, in the case of the Diffusion process, the execution time depends not only on the number of nodes but also on the edges between the nodes since the Diffusion process calculates the scores for all the edges in the given subgraph.

**Figure 4:**
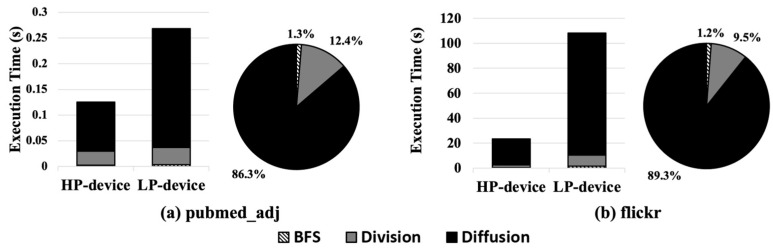
Execution time breakdown of each subprocess (BFS, Division, and Diffusion) in PPR depending on the size of graph dataset.

Figure 4 shows the execution time and the proportion of each subprocess (*BFS*, *Division*, and *Diffusion*) when executing a PPR with a small graph dataset, *pubmed_adj* (Figure 4a), and a large graph dataset, *flickr*, (Figure 4b); the details on the dataset will be explained in Section 4.1.

In the case of pubmed_adj (Figure 4a), due to the small size of the graph, the execution time of the BFS and Division process is not much different between the HP and LP edge devices, while that of the Diffusion process shows a significant difference in execution time between the devices. On the other hand, in the case of flickr (Figure 4b), which is much larger than pubmed_adj, the execution time increases significantly (>5×) in the LP edge device compared to the HP edge device. The reason for this is that the larger graph causes a longer execution time in the Division and Diffusion processes, whereas it results in a negligible difference in the BFS.

According to our analysis, the characteristics of the PPR subprocesses can be described as follows.

1. The BFS accounts for a negligible portion of the execution time of the PPR. Thus, it would be better to execute BFS on the LP edge device. Furthermore, it could be better to execute BFS on CPUs, not on GPUs, since the GPU execution causes memory copy (e.g., cudamemcpy) and kernel launch overheads.
2. The Division process has variable characteristics in execution time depending on the size of graph. In the case of a small graph size, such as pubmed_adj (Figure 4a), the difference in the Division execution time is marginal. However, in the case of a large graph size, such as flickr (Figure 4b), the Division process suffers from non-negligible performance degradation in the LP edge device.
3. The Diffusion process constitutes the largest portion of the execution time in PPR, regardless of whether in HP or LP edge devices, because it executes arithmetic operations for the nodes and edges in the subgraph. Thus, Diffusion should be executed on the GPUs of the HP device for better performance and energy efficiency.

**Figure 5:**
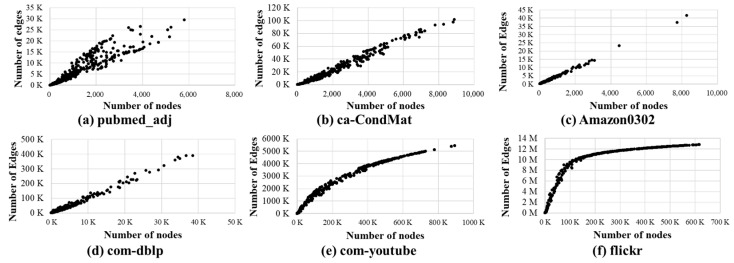
Analysis of the correlation between the number of nodes (*x*-axis) and edges (*y*-axis) in subgraphs for various graph datasets.

Figure 5 shows the analysis of the relationship between the number of nodes and edges in subgraphs when constructing subgraphs using BFS and the Division process using the SNAP dataset [26].

As shown in Figure 5, the number of edges is typically proportional to the number of nodes in subgraphs; note that the correlation coefficient is between 0.93~0.99. As the number of edges and nodes increases, the execution time of Division and Diffusion could be longer. According to this observation, it is possible to accurately decide which edge device will be efficient to execute Division or Diffusion for each subgraph based on the BFS for each second PPR [16]. Our proposed *HedgeRank* provides an energy-efficient partitioning with this methodology.

### 3.2. Overview of HedgeRank

**Figure 6:**
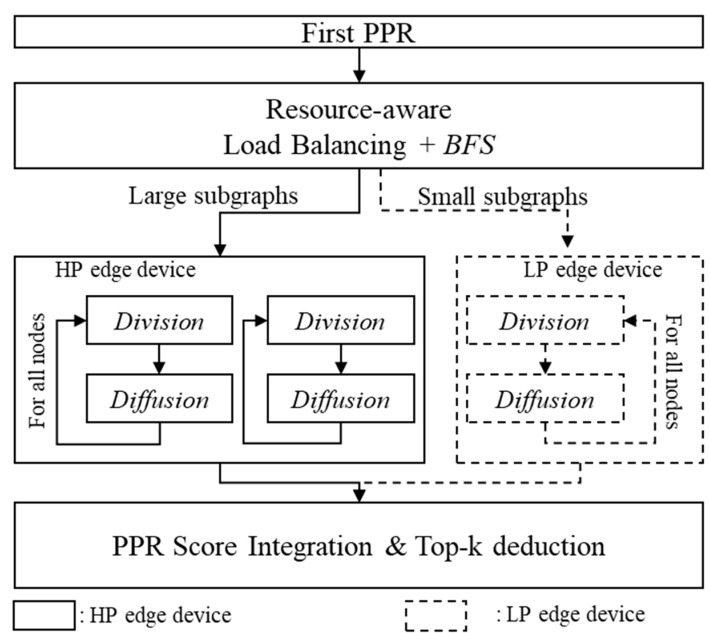
Flow chart diagram of *HedgeRank* with the low-power (LP) and high-performance (HP) edge device configuration.

Figure 6 represents the overall flow chart of *HedgeRank*.

First, *HedgeRank* computes the PageRank scores on a subgraph within the distance l1 from the starting node and calculates the first PPR to find the top-k nodes (i.e., the next-stage nodes). Next, to distribute the second PPR calculations considering the resource ratio of edge devices, BFS is executed within the distance l2 for all the next-stage nodes. According to the results from the BFS, we are able to obtain the number of nodes participating in the second PPR. In addition, the calculation overhead in Division and Diffusion can be predicted by the number of nodes in the subgraphs. Thus, we can distribute the subgraphs into HP or LP edge devices by considering the resources of the edge devices. Finally, each edge device computes Division and Diffusion for the assigned subgraphs. The computed scores are gathered on the high-performance edge device to determine the top-k nodes.

Division can be calculated independently for each node, so it is computed using a single thread per node. As a result, a subgraph can be constructed for the connected nodes. Diffusion can be calculated independently for each edge, so Diffusion for each edge is computed using a single thread, which leads to the new scores for each edge. Lastly, based on the Diffusion result, the final score of each node can be computed using a thread per node.

Algorithm 1 presents the resource-aware load balancing of *HedgeRank*, which distributes the subgraph computation to edge devices depending on the size of the subgraphs and the resource ratio.
**Algorithm 1** Resource-aware Load Balancing**Input:** Graph *G*, Next–stage node vector *N*, Resource ratio *R***Output:** Subgraph list on LP device *L*, Subgraph list on HP device *H*1: *vector n*                                                             ▷ Nodes found in BFS2: *s* = 0                                                                  ▷ Sum of the nodes3: **for each**
*i ∈ N*
**do**                                           ▷ BFS for each node4:          *n*[*i*] = *BFS*(*i, G*)5:          *s* += *n*[*i*]*.size*6: **end for**7: *Ascending_Order_Sort(N, n)*8: **for each**
*i ∈ N*
**do**                                           ▷ Resource-aware Load Balancing9:            **if**
*s/R >*= *L.size* + *n*[*i*]*.size*
**then**10:               *L* += *n*[*i*]                                            ▷ Assign the node to LP device11:          **else**12:                *H* += *n*[*i*]                                           ▷ Assign the node to LP device13:          **end if**14: **end for**

First, to predict the amount of computation for the subgraph, BFS is performed for all the next-stage nodes. Then, all the next-stage nodes are sorted by ascending order. Finally, starting from the next-stage nodes with fewer nodes in the subgraph, the resource ratio is calculated by reflecting the computational capability of the given edge devices, which corresponds to the core function of resource-aware load balancing. Based on the resource-aware load balancing, *HedgeRank* assigns each subgraph to an appropriate edge device, improving energy efficiency as well as performance.

In our resource-aware load balancing methodology, we use the number of GPU cores as a metric to determine the resource ratio. The distribution of computations was carried out by assigning the subgraphs with the smallest number of nodes to LP edge devices while ensuring the number of nodes to be calculated does not exceed the resource ratio (as shown in line No. 9 in Algorithm 1). When the LP edge device exceeds its computation capability, the remaining subgraphs are assigned to HP edge devices.

For example, assuming that a LP edge device has 128 GPU cores and a high-performance edge device has 512 GPU cores, the resource ratio is set to 5 to allocate computations at a 1:4 ratio. With the resource ratio of 5, starting from the smallest subgraph, the computations are assigned to low-power edge devices until the total number of nodes in the assigned subgraphs exceeds 1/5 of the total number of nodes in all the subgraphs, and the remaining computations are assigned to high-performance edge devices. To reduce end-to-end latency from using devices with different specifications, load balancing while considering the hardware resources is important. By distributing and allocating computations to each edge device using the method we propose, execution time is reduced due to the proper PPR parallelization among edge devices, and as mentioned in Section 2, low-power edge devices consume much less energy than high-performance edge devices when processing smaller subgraphs, which can reduce the energy required during PPR execution.

## 4. Evaluation

### 4.1. Experimental Environment

**Figure 7:**
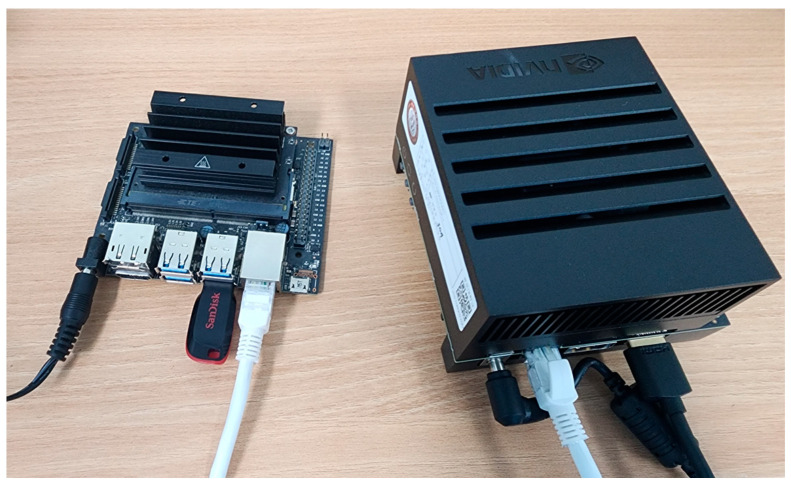
A photograph of our experimental environment with Nvidia Jetson Nano (**left**) and AGX Xavier (**right**).

Figure 7 shows a photograph of our experimental environment with real-world edge devices. Table 1 shows the specifications of the edge devices used in our experiment. For the high-performance (HP) edge device, we use a NVIDIA Jetson AGX Xavier [25] with the maximum power budget (30 W). Furthermore, we consider the mid-range performance (MP) edge device by limiting the power budget (= 10 W) of the Jetson AGX Xavier; the MP edge device has lower voltage and frequency than the HP edge device. Lastly, we use NVIDIA Jetson Nano [27] as a low-power edge device (LP device). Based on the edge devices shown in Table 1, we analyze *HedgeRank* for three configurations: (1) LP + HP, with a 1:4 resource ratio, (2) LP + MP, with 1:2 resource ratio, and (3) MP + HP, with 1:2 resource ratio. We compare *HedgeRank* to MeLoPPR using single-device-only configurations (e.g., LP-only, MP-only, and HP-only); please note that we modified the original MeLoPPR to a GPU-optimized version for a fair comparison, while MeLoPPR uses only CPU in the original version [16].

Using the edge devices, we analyze the execution time and energy consumption. For execution time, we breakdown the execution time of the PPR based on the NVIDIA Nsight Systems profiler. Since the Nvidia Nsight Systems profiler is executed on the host (server) to profile the execution time of processes/threads (CPU threads as well as GPU kernels) running on the connected edge devices, it can be used to measure the execution time accurately without incurring any overhead (i.e., noise) in execution time. Furthermore, we measure the voltage and current of the components (e.g., CPU, GPU, SoC, memory, etc.) in the edge devices based on the Jetson API provided by Nvidia; note that the real measurement methodology has been widely used in previous studies with Nvidia Jetson series [28,29]. We calculate the power consumption by using the measured voltage and current values. To summarize, by measuring the execution time and power consumption, we analyze the total energy consumption (Execution time × Power) of PPR on edge devices, and we compare the energy efficiency of *HedgeRank* to that of MeLoPPR.

Table 2 shows the characteristics of the graph datasets used in our evaluation. We used six various-sized graphs from the SNAP dataset [26]. To evaluate our proposed *HedgeRank*, we considered MeLoPPR as the baseline. For both, the total PPR length, *L*, was set to six, *l*1 and *l*2 were set to three, and the number of next-stage nodes was set to 5. We conducted 100 experiments for each case by selecting the starting node randomly for each iteration. We measured the execution time and power consumption of the edge devices.

### 4.2. Execution Time Analysis

**Figure 8:**
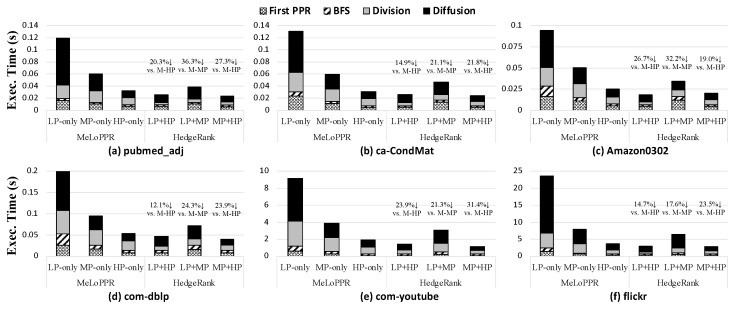
Execution time of *HedgeRank* with three different configurations (LP + HP, LP + MP, and MP + HP) compared to MeLoPPR with each single device-only configuration (LP-only, MP-only, and HP-only). Note M-xP stands for MeLoPPR with xP-only configuration.

Figure 8 shows the execution time of *HedgeRank* for three different configurations compared to that of MeLoPPR; in the Figure 8, x% ↓ indicates execution time reduction, while the x% ↑ means execution time increase. As shown in Figure 8, for a relatively small graph dataset, such as *pubmed_adj* and ca-CondMat (Figure 8a,b, respectively), the performance difference between the different edge devices is not that significant as they have fewer nodes and edges. Nevertheless, in the case of *pubmed_adj* (Figure 8a), *HedgeRank* (LP + HP) improves the execution time by 20.3% compared to MeLoPPR with HP-only (denoted as M-HP in Figure 8). On the other hand, the large graph datasets such as *com-youtube* and *flickr* (Figure 8e and f, respectively) have a much larger number of nodes and edges than the others. Thus, such a large graph would be processed faster in the HP-device leading to better performance. As a result, in the case of *flickr* (Figure 8f), *HedgeRank* (LP + HP) reduces the execution time by 14.6% compared to M-HP. In all the cases, *HedgeRank* (LP + HP) outperforms M-HP as it efficiently partitions subgraphs into different edge devices based on our resource-aware load balancing. In summary, *HedgeRank*, with LP + HP devices, provides a 12.1%~26.7% shorter execution time than MeLoPPR with HP only. Meanwhile, *HedgeRank* must be better than MeLoPPR because our *HedgeRank* exploits two different devices for PPR execution while MeLoPPR only uses a single device. More importantly, *HedgeRank* reduces energy consumption compared to MeLoPPR as it distributes the PPR subprocess to the different edge devices by considering their computation capabilities and graph sizes.

### 4.3. Energy Consumption Analysis

**Figure 9:**
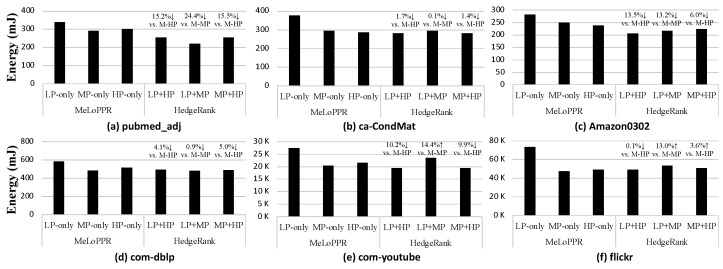
Energy consumption of *HedgeRank* with three different configurations (LP + HP, LP + MP, and MP + HP), compared to MeLoPPR with each single device-only configuration (LP-only, MP-only, and HP-only). Note M-xP stands for MeLoPPR with xP-only.

Figure 9 shows the energy consumption when running the *HedgeRank* (for LP and MP, LP + HP, MP + HP) and the GPU-optimized MeLoPPR on each single device; in the Figure 8, x% ↓ indicates energy reduction, while the x% ↑ means energy overhead. As shown in Figure 9, *HedgeRank* (LP + HP) reduces the energy consumption of edge devices in all the graph datasets, even with better performance, as described in the previous subsection. For example, in the case of *pubmed_adj* (Figure 9a), *HedgeRank* (LP + HP) provides a 15.2% lower energy consumption compared to the MeLoPPR with HP-only, while it provides a 20.3% lower execution time. In the case of *flickr* (Figure 9f), which is the largest graph in our evaluation, *HedgeRank* (LP + HP) shows similar energy consumption compared to MeLoPPR with HP-only, while it improves execution time by 14.7%. Though *HedgeRank* (LP + HP) provides better energy consumption as well as execution time than MeLoPPR with HP-only, *HedgeRank* with an MP (i.e., LP + MP or MP + HP) device could have a higher energy consumption than MeLoPPR. According to our analysis, *HedgeRank* would be effective to improve energy efficiency if it is applied to two edge devices with a large resource difference (e.g., computation capability, memory, etc.). Since the LP + MP or MP + HP configurations have smaller differences in their computation capabilities and memory capacity compared to the LP + HP configuration, *HedgeRank* may have a higher energy consumption than MeLoPPR; note, however, that it always provides a better performance.

### 4.4. Overhead Analysis

Table 3 shows the subgraph size partitioned by *HedgeRank* in the LP + HP configuration. The resource ratio of the LP + HP configuration is determined as being 1:4 by considering the difference in computation capability. As shown in Table 3, the subgraph size assigned to the LP-device is of the order of several bytes to hundreds of kilobytes. Due to the small size of the subgraphs, the data transfer overhead between HP device and LP device is not significant; note that we assume that the first PPR and resource-aware load balancing are executed on the HP-device.

## 5. Summary and Discussion

**Summary of HedgeRank**: There have been many studies exploiting graph processing such as personalized PageRank (or PageRank). However, among them, only a few studies focused on the acceleration of PPR itself with a hardware/software co-design. We summarized the state-of-the-art PPR acceleration techniques compared to *HedgeRank* in Table 4. Though MeLoPPR was published in 2021, we selected MeLoPPR as our baseline because MeLoPPR is the state-of-the-art study aiming to improve PPR without special hardware such as PIM. Compared to the MeLoPPR, the most advantageous feature of our proposed *HedgeRank* is the edge availability. *HedgeRank* does not even require FPGA implementation but only uses the general processing units CPU (C++), GPU (CUDA), or both. Though PIM/FPGA implementation could be better than pure software implementation for a specific size of graph, it needs to be redesigned when the characteristics of the graphs becomes different. In real-world applications, various graphs have significantly different characteristics, as we also described in our analysis. Additionally, graphs can be changed dynamically at runtime, which requires reconfigurability and scalability for PPR acceleration techniques. In terms of reconfigurability and scalability, processing-in-memory (PIM)-based graph accelerators also have critical weaknesses due to their optimized and specialized hardware designs. On the other hand, *HedgeRank* can be executed on edge devices with various graphs without modifications as it is implemented with pure software (C++ and CUDA) implementation.

**Impact of Network Conditions:** Though *HedgeRank* requires data transfer between edge devices, the size of data to be transferred is quite small (only a few hundred kilobytes even in large graphs), as described in Section 4.4. Thus, the network impact would be negligible. If we use cellular networks such as 5G, not an Ethernet interface or Wi-Fi, we need to carefully consider network conditions such as signal strength and modem chip power consumption for partitioning PPR to different edge devices. Though we did not include these complicated network considerations in this study, we plan to extend our study to real mobile devices with cellular networks.

**Potential Application of *HedgeRank***: Thanks to the benefits of *HedgeRank*, it is expected to be applied to various applications exploiting PPR for pre-/post-processing. For example, in the case of recommender systems [23], PPR has been widely used for filtering user-aware or context-aware features on the whole graph data to provide better recommendations. In this case, PPR-assisted recommendation models could be executed with better energy efficiency by using *HedgeRank* on edge devices since *HedgeRank* partitions the whole graph data considering the graph data characteristics as well as the hardware resource constraints. Furthermore, since *HedgeRank* does not require any modification/addition of the original graph data, it may be orthogonally applied to other techniques to reduce PPR computations.

## 6. Conclusions

In this study, we propose *HedgeRank*, an energy-efficient personalized PageRank (PPR) partitioning technique for a heterogeneous edge environment. *HedgeRank* takes into account computation capability and the size of subgraphs for energy-efficient PPR execution for edge environments. According to our analysis, *HedgeRank* provides better energy efficiency as well as a shorter execution time than the state-of-the-art PPR technique. In the case of *HedgeRank* with a low-power (LP) and a high-performance (HP) edge device configuration (LP + HP), it provides lower execution time (12.1%~26.7%), with lower energy consumption (0.1%~15.2%) compared to the state-of-the-art PPR technique, MeLoPPR. Our *HedgeRank* could be orthogonally applied to many applications with PPR, such as various algorithms based on graph processing [6] as well as graph neural networks (GNNs) [5]. We expect that *HedgeRank* will be applied for a wider range of applications for edge environments for better energy efficiency without sacrificing performance.

## Figures and Tables

**Table 1:** Specifications of edge devices used in our evaluation.

	Nvidia Jetson AGX Xavier 30 W (HP Device)	Nvidia Jetson AGX Xavier 10 W (MP Device)	Nvidia Jetson Nano (LP Device)
CPU	8-cores ARM Carmel v.8.2 @ 2.26 GHz	2-cores ARM Carmel v.8.2 @ 1.2 GHz	4-cores ARM A57 @ 1.43 GHz
GPU	Volta 512 cores @ 1.4 GHz	Volta 256 cores @ 512 MHz	Maxwell 128 cores @ 537 MHz
Memory	32 GB of LPDDR4x ~2133 MHz	32 GB of LPDDR4x ~1066 MHz	4 GB of LPDDR4 ~1600 MHz
Power	30 W	10 W	10 W

**Table 2:** Graph datasets used in our evaluation.

Graph Name	Number of Nodes	Number of Edges
*pubmed_adj*	19.717 K	44.327 K
*ca_CondMat*	23.133 K	93.497 K
*Amazon0302*	262,111 K	1234.877 K
*com-dblp*	317.080 K	1049.866 K
*com-youtube*	1134.890 K	2987.624 K
*flickr*	820.878 K	9837.214 K

**Table 3:** Subgraph size partitioned by *HedgeRank* in the LP + HP configuration.

Graph Name	HP Device	LP Device	Sum
*pubmed_adj*	5.1 KB	1.8 KB	6.9 KB
*ca_CondMat*	9.5 KB	2.7 KB	12.2 KB
*Amazon0302*	8.4 KB	0.655 KB	9.1 KB
*com-dblp*	21.2 KB	3.7 KB	25.0 KB
*com-youtube*	1783.5 KB	357.7 KB	2141.2 KB
*flickr*	1345.0 KB	259.4 KB	1604.4 KB

**Table 4:** Comparison of the state-of-the-art PPR acceleration techniques and HedgeRank.

Parameter	Graphicionado [18]	Graphh, GraphR [9,19]	MeLoPPR [16]	HedgeRank (Our Work)
Date of publication	2016	2018	2021	2023
Hardware	PIM	PIM	CPU, FPGA	CPU, GPU
Extensibility (e.g., NN algorithms)	△ (Need of PIM API)	△ (Need of PIM API)	△ (Need of FPGA Impl.)	O
Reconfigurability	X	X	△ (Need of re-design)	O
Scalability	X	X	△ (Need of more LUTs)	O
Edge Availability	X	X	X	O

## Data Availability

Data are contained within the article. For more details, please contact the corresponding author.
